# Global Changes and Factors of Increase in Caloric/Salty Food Intake, Screen Use, and Substance Use During the Early COVID-19 Containment Phase in the General Population in France: Survey Study

**DOI:** 10.2196/19630

**Published:** 2020-09-18

**Authors:** Benjamin Rolland, Frédéric Haesebaert, Elodie Zante, Amine Benyamina, Julie Haesebaert, Nicolas Franck

**Affiliations:** 1 Service Universitaire d'Addictologie de Lyon CH Le Vinatier Hospices Civils de Lyon Bron France; 2 Inserm U1028, CNRS UMR5292 Université Claude Bernard Lyon 1 Bron France; 3 Centre Ressource de Réhabilitation Psychosociale CH Le Vinatier Lyon France; 4 PSYCOMADD 4872 Université Paris-Saclay Paris France; 5 CH Le Vinatier Pôle Centre Rive Gauche Bron France; 6 Service de recherche et d’épidémiologie Pôle de santé publique Hospices Civils de Lyon Lyon France

**Keywords:** COVID-19, containment, eating behaviors, screen use, internet use, substance use, public health, mental health, pandemic, lifestyle, online survey, addiction

## Abstract

**Background:**

The international outbreak of coronavirus disease (COVID-19) has led many countries to enforce drastic containment measures. It has been suggested that this abrupt lockdown of populations will foster addiction-related habits such as caloric/salty food intake, screen use, and substance use.

**Objective:**

Our aim was to assess the global changes and factors of increase in addiction-related habits during the early COVID-19 containment phase in France.

**Methods:**

A web-based survey was provided from day 8 to day 13 of the containment and was completed by 11,391 participants. The questions explored sociodemographic features, psychiatric/addiction history, material conditions of lockdown, general stress, mental well-being, and reported changes in several addiction-related behaviors. Global changes were described and factors of increase were explored using population-weighted and adjusted logistic regression models, providing adjusted odds ratios (aORs) and their 95% confidence intervals.

**Results:**

Overall, the respondents reported more increases in addiction-related habits than decreases, specifically 28.4% (caloric/salty food intake), 64.6% (screen use), 35.6% (tobacco use), 24.8% (alcohol use), and 31.2% (cannabis use). Reduced well-being scores and increased stress scores were general factors of increase in addiction-related habits (*P*<.001 for all habits). Factors of increase in caloric/salty food intake (n=10,771) were female gender (aOR 1.62, 95% CI 1.48-1.77), age less than 29 years (*P*<.001), having a partner (aOR 1.19, 95% CI 1.06-1.35), being locked down in a more confined space (per 1 square meter/person decrease: aOR 1.02, 95% CI 1.01-1.03), being locked down alone (aOR 1.29, 95% CI 1.11-1.49), and reporting current (aOR 1.94, 95% CI 1.62-2.31) or past (aOR 1.27, 95% CI 1.09-1.47) psychiatric treatment. Factors of increase in screen use (n=11,267) were female gender (aOR 1.31, 95% CI 1.21-1.43), age less than 29 years (*P*<.001), having no partner (aOR 1.18, 95% CI 1.06-1.32), being employed (*P*<.001), intermediate/high education level (*P*<.001), being locked down with no access to an outdoor space (aOR 1.16, 95% CI 1.05-1.29), being locked down alone (aOR 1.15, 95% CI 1.01-1.32), living in an urban environment (*P*<.01), and not working (*P*<.001). Factors of increase in tobacco use (n=2787) were female gender (aOR 1.31, 95% CI 1.11-1.55), having no partner (aOR 1.30, 95% CI 1.06-1.59), intermediate/low education level (*P*<.01), and still working in the workplace (aOR 1.47, 95% CI 1.17-1.86). Factors of increase in alcohol use (n=7108) were age 30-49 years (*P*<.05), a high level of education (*P*<.001), and current psychiatric treatment (aOR 1.44, 95% CI 1.10-1.88). The only significant factor of increase in cannabis use (n=620) was intermediate/low level of education (*P*<.001).

**Conclusions:**

The early phase of COVID-19 containment in France led to widespread increases in addiction-related habits in the general population. Reduced well-being and increased stress were universal factors of increase. More specific factors were associated with increases in each of the explored habits.

## Introduction

In March 2020, the outbreak of coronavirus disease (COVID-19) led the national authorities of most countries worldwide to implement extraordinary measures that dramatically restricted the mobility and social interactions of their populations with the aim of limiting transmission of the virus [1]. In this respect, many countries, including Italy, France, and Spain, decided to establish total or at least very strict lockdown. In France, this containment was announced by the President on March 16, and it went into effect at noon ECT on March 17, 2020 [2]. Only activities deemed “essential” were maintained; these included some medical activities but also activities related to the food supply, including access to alcohol as well as to tobacco and electronic cigarette shops. In France, as in other countries, due to this unprecedented situation, a large majority of the population became locked down at home overnight.

These containment measures, as well as the abrupt international health and economic crises caused by COVID-19, may have caused substantial stress in the population and thus may have significantly impacted people’s general health and, more specifically, their mental well-being. Previous situations of reduced well-being and impaired social environment have been found to be associated with overeating and being overweight as well as increased substance and screen use [3-5]. In this context, it has been suggested that at-risk behaviors that are in the spectrum of addiction are likely to be exacerbated by the COVID-19 outbreak and the related containment but that this should be confirmed by studies [6].

The LockUwell study is a nationwide web-based survey aiming to assess the overall effects of the official containment on the French general population with respect to mental well-being and general health conditions, including eating habits as well as screen and substance use. In this study, we describe the containment-related changes in the respondents’ daily habits of eating, screen use, and substance use, and we explore the main characteristics of the participants who reported the most substantial changes.

## Methods

### Type of Study

An open web-based survey was launched on March 25, 2020, that is, 8 days after the official implementation of the containment in France.

The reporting of the survey follows the Checklist for Reporting Results of Internet E-Surveys (CHERRIES) [7]. The completed checklist can be found in Multimedia Appendix 1.

### Recruitment

Any French-speaking person older than 16 years was invited to participate in the survey without restriction criteria provided they could complete the questionnaire autonomously. The link leading to the online survey was disseminated on the internet using social media (ie, Twitter, LinkedIn, and Facebook) and national media. The recruitment strategy thus followed a convenience sampling method. To prevent individuals from completing the questionnaire multiple times, only one questionnaire could be submitted from a particular IP address.

### Questionnaire

The English version of the full questionnaire is available in Multimedia Appendix 2. There was no preliminary assessment of the test-retest reliability or the internal consistency of the questionnaire; however, several tools included in it were previously validated in international studies.

The survey questions aimed to comprise a large range of items related to mental well-being and psychological distress and to collect sociodemographic and environmental data related to the situation of containment, such as total living space or number of persons sharing the house during the containment. The questionnaire was divided in six consecutive sections: 1) sociodemographic features; 2) the French version [8] of the Warwick-Edinburgh Mental Well-Being Scale (WEMWBS), which is a validated scale of 14 items that are rated from 1 to 5, leading to a single total score ranging from 14 (ie, minimum possible well-being) to 70 (ie, maximum possible well-being) to measure mental well-being in the general population [9]; 3) overall and specific (eg, professional, health-related, family) levels of stress, using a visual analog scale ranging from 0 (no stress) to 10 (maximum possible stress) [10]; 4) medical history, in particular the history of psychiatric and addiction treatment; 5) perceptions and apprehensions about COVID-19 and the related official measures; and 6) personal and environmental conditions under which participants were facing the lockdown and their consequences.

In particular, question F-25 explored whether respondents had changed their intake of caloric/salty food, their use of screens, and their use of substances (tobacco, alcohol, cannabis, and other drugs). The response modalities were 1) no usual use; 2) no change in use; 3) decrease with craving/withdrawal; 4) decrease without craving/withdrawal; 5) increase (moderate); and 6) increase (difficult to control).

### Data Extraction and Preprocessing

The data were extracted on March 30, 2020, that is, 5 days after the start of the survey. For the present analysis, we included only respondents aged 16 years and older who completed the questionnaire and were living in France at the time when containment was declared. Among the 20,235 participants who started the questionnaire, 11,742 (58.0%) completed it. After excluding inoperable questionnaires and respondents from countries other than France, 11,391/20,235 questionnaires (56.3%) were included in the analyses. A complete flowchart is displayed in Multimedia Appendix 3. Only the responses to questions A-1 to A-7, A-12 to A-14, A-16, B-1, C-1c, D-4b and D-4c, F-6 to F-9, F-17, and F-25 were used in this preliminary investigation.

### Statistical Analysis

Statistical analyses were performed using SAS software version 9.4 (SAS Institute). To ensure respondents were representative of French residents aged 16 years and older, the data were weighted to French census targets for age and gender based on distributions reported in 2020 [11]. All descriptive and statistical tests were conducted using weighting variables. The descriptive statistics display categorical variables as the number and percentage of respondents (n, %), while quantitative variables are presented as mean (SD) or median (IQR). We explored caloric/salty food intake, screen use, and tobacco, alcohol, and cannabis use because insufficient data were collected regarding other substances. For each behavior and substance used, the different levels of subjective change (ie, no change, decrease with craving/withdrawal, decrease without craving/withdrawal, moderate increase, or difficult-to-control increase) are displayed.

Increase was expected to be a much more frequent pattern of change than decrease for all behaviors; therefore, we more deeply explored the parameters specifically associated with increase (both types of increase combined) in each type of behavior compared to other modalities of change using weighted logistic regression models. Respondents declaring no usual use were not included in the analyses. For each model, raw odds ratios (ORs) and adjusted odds ratios (aORs) are provided with their 95% confidence intervals. We entered the following variables in the model (each was adjusted with the others in the adjusted analyses): sociodemographic factors (age, gender, family, occupation, and educational level), psychiatric and addiction history, well-being (WEMWBS total score), stress (general stress VAS), housing conditions (surface, outdoor, geographical area) and working conditions during containment as explanatory variables. Multicollinearity was screened using the variance inflation factor and the COLLIN option in SAS.

## Results

The raw and weighted descriptive data of the 11,391 participants are shown in Table 1. The weighted sample consisted of 52.1% female respondents with a mean age of 47.47 years (SD 17.28).

**Figure 1:**
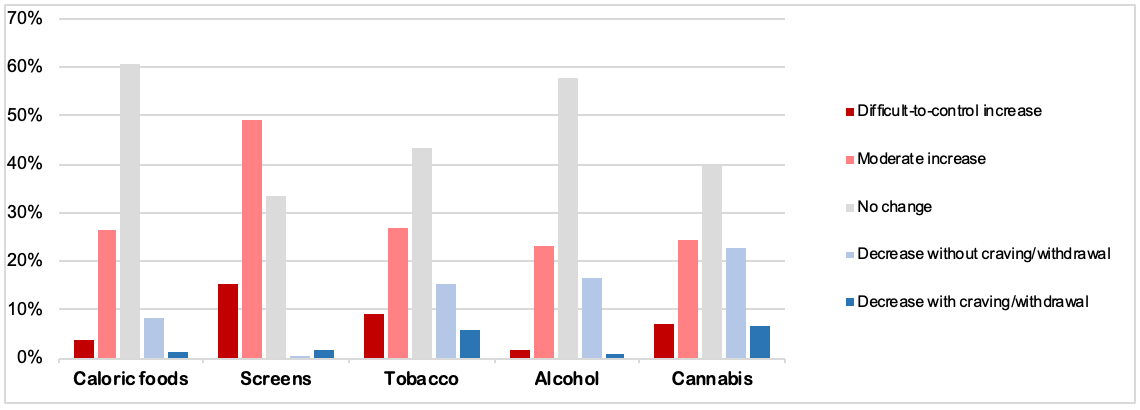
Changes in addiction-related habits in the early phase of COVID-19 containment in France from March 17 to 31, 2020.

The overall changes reported in the daily habits that were explored in the survey are displayed in Figure 1.

**Table 1:** Descriptive characteristics of the survey population (N=11,391).

Characteristic		Values	
		Unweighted	Weighted
**Age (years), mean (SD)**			
	16-29	3404 (29.88)	2421 (21.26)
	30-49	5316 (46.67)	3488 (30.61)
	50-64	2043 (17.94)	2651(23.27)
	65-74	547 (4.80)	2469 (21.67)
	≥75	81 (0.71)	364 (3.20)
**Gender, n (%)**			
	Male	2557 (22.45)	5415 (47.5)
	Female	8782 (77.10)	5932 (52.1)
	Other	52 (0.46)	52 (0.4)
**Marital status, n** **(%)**			
	Single, divorced, or widowed	4033 (35.41)	4215 (37)
	In a couple	7358 (64.59)	7178 (63)
**Employment status, n** **(%)**			
	Worker	8032 (70.51)	6486 (56.92)
	Job seeker	568 (4.99)	475 (4.17)
	Student	1407 (12.35)	987 (8.66)
	No employment or retired	1384 (12.15)	3447 (30.25)
**Educational level (ISCED^a^ 2011), n (%)**			
	≤3	727 (6.38)	1074 (9.42)
	4	1326 (11.64)	1485 (13.03)
	5-6	3985 (34.98)	3727 (32.71)
	≥6	5353 (46.99)	5108 (44.83)
**Psychiatric history, n** **(%)**			
	Current	1244 (10.92)	1031 (9.05)
	Past	1632 (14.33)	1622 (14.24)
	Never	8515 (74.75)	8740 (76.71)
**Addiction treatment, n** **(%)**			
	Current	78 (0.68)	80 (0.71)
	Past	223 (1.96)	286 (2.51)
	Never	11090 (97.36)	11026 (96.78)
**Access to outdoor space, n** **(%)**			
	Yes	6911 (60.67)	7103 (62.34)
	No	4480 (39.33)	4291(37.66)
Housing space (square meters/person), median (IQR)		34.67 (25-50)	40.00 (28-60)
Well-being (WEMWBS^b^ score), mean (SD)		49.37 (8.12)	50.51 (8.17)
General stress (0-10 VAS^c^), mean (SD)		5.23 (2.35)	4.84 (2.43)
**Housing location, n** **(%)**			
	Urban	6303 (55.33)	6375 (55.95)
	Periurban	2419 (21.24)	2409 (55.95)
	Rural	2669 (23.43)	2610 (55.95)
**People locked down in the household (including the respondent), n** **(%)**			
	1	2528 (22.20)	3159 (27.73)
	≥2 but <10	8845 (77.66)	8214 (72.10)
**Work location during lockdown, n** **(%)**			
	In the workplace	2266 (19.89)	1755 (15.41)
	Telecommuting	4708 (41.33)	3871 (33.97)
	Not working	4417 (41.33)	5768 (50.62)
**Change in caloric/salty food intake, n** **(%)**			
	No intake	511 (4.49%)	622 (5.50)
	No change	5655 (49.64%)	6510 (57.14)
	Increase	4125 (36.21%)	3233 (28.38)
	Decrease	1100 (9.65%)	1028 (9.02)
**Change in screen use, n** **(%)**			
	No use	99 (0.87)	127 (1.11%)
	No change	3241 (28.45)	3785 (33.22)
	Increase	7843 (68.85)	7274 (63.84)
	Decrease	208 (1.82)	208 (1.82)
**Change in tobacco use, n** **(%)**			
	No use	8241 (72.35)	8607 (75.55)
	No change	1218 (10.69)	1208 (10.55)
	Increase	1279 (11.23)	995 (8.74)
	Decrease	653 (5.74)	589 (5.17)
**Change in alcohol use, n** **(%)**			
	No use	4292 (37.68)	4285 (37.62)
	No change	3708 (32.55)	4109 (36.07)
	Increase	2023 (17.76)	1761 (15.46)
	Decrease	1368 (12.0)	1237 (10.86)
**Change in cannabis use, n** **(%)**			
	No use	10697 (93.91)	10724 (94.12)
	No change	264 (2.32)	263 (2.31)
	Increase	233 (2.05)	210 (1.84)
	Decrease	197 (1.73)	195 (1.73)

^a^ISCED: International Standard Classification of Education.

^b^WEMWBS: Warwick-Edinburgh Mental Well-Being Scale.

^c^VAS: visual analog scale.

Regarding eating patterns, 6510/11,391 (57.14%) participants reported that they did not increase or decrease their average daily intake of caloric/salty food, whereas 2836 (24.89%) moderately increased their intake, 397 (3.49%) increased their intake in a difficult-to-control manner, 874 (7.67%) reduced their intake without craving, and 154 (1.35%) reduced their intake with craving.

With respect to screen use, 124/11,391 (1.09%) respondents declared that they did not usually use screens. Among the 11,267 remaining participants, 3784 (33.59%) reported that they did not change their average daily screen use, whereas 5545 (49.22%) declared having moderately increased their screen use, 1729 (15.35%) increased their screen use in a difficult-to-control manner, 179 (1.59%) reduced or stopped their screen use without craving/withdrawal, and 29 (0.26%) reduced their screen use with craving/withdrawal.

Concerning tobacco use, 2787/11,391 (24.47%) respondents reported that they were current smokers. Among the 2787 smokers, 1208 (43.27%) reported that they did not change their average daily use of tobacco, whereas 746 (26.72%) declared having moderately increased their tobacco use, 249 (8.92%) increased their tobacco use in a difficult-to-control manner, 432 (15.47%) declared that they reduced or stopped their tobacco use without craving/withdrawal, and 157 (5.62%) reduced their tobacco use with craving/withdrawal. Regarding alcohol use, 7108/11,391 (62.40%) respondents were found to use alcohol more or less regularly. Among them, 4109/7108 (57.82%) reported that they had not changed their average daily use of alcohol, whereas 1654 (23.27%) moderately increased their alcohol use, 107 (1.50%) increased their alcohol use in a difficult-to-control manner, 1167 (16.4%) declared having reduced or stopped without craving/withdrawal, and 70 (0.98%) having reduced with craving/withdrawal.

Finally, regarding cannabis use, 620/11,391 (5.44%) participants reported using cannabis. Among the, 263/620 (39.49%) reported that they had not changed their average daily use of cannabis, whereas 162 (24.32%) declared having moderately increased their cannabis use, 46 (6.91%) increased their cannabis use in a difficult-to-control manner, 150 (22.52%) reduced or stopped their cannabis use without craving/withdrawal, and 45 (6.76%) reduced their cannabis use with craving/withdrawal.

Raw and adjusted analyses of the factors associated with the increase in each of the explored habits can be found in Table 2 and Table 3, respectively.

**Table 2:** Results of the unadjusted analyses exploring the increases in caloric/salty food, screen use, and substance use in the early phase of COVID-19 containment in France among the general population.

Characteristic		Caloric/salty food intake (n=10,771; 3233 increase vs 7538 no increase)				Screen use (n=11,267; 7274 increase vs 3993 no increase)				Tobacco use (n=2787; 996 increase vs 1791 no increase)					Alcohol use (n=7108; 1761 increase vs 5347 no increase)					Cannabis use (n=666; 208 increase vs 458 no increase)				
		OR^a^ (95% CI)		*P* value		OR (95% CI)		*P* value			OR (95% CI)		*P* value			OR (95% CI)		*P* value			OR (95% CI)		*P* value	
**Gender**																								
	Male		Reference		N/A^b^		Reference		N/A			Reference		N/A			Reference		N/A			Reference		N/A
	Female		1.59 (1.46-1.73)		<.001		1.33 (1.23-1.44)		<.001			1.36 (1.16-1.59)		<.001			1.13 (1.12-1.26)		.02			1.01 (0.71-1.44)		.96
	Other		2.08 (1.17-3.68)		.01		1.57 (0.86-2.87)		.14			1.26 (0.46-3.50)		.67			0.53 (0.19-1.54)		.25			0.63 (0.13-3.05)		.57
**Age (years)**																								
	16-29		Reference		N/A		Reference		N/A			Reference		N/A			Reference		N/A			Reference		N/A
	30-49		0.85 (0.77-0.95)		<.001		0.61 (0.54-0.69)		<.001			0.89 (0.73-1.08)		.25			1.33 (1.16-1.53)		<.001			0.90 (0.63-1.28)		.53
	50-64		0.50 (0.44-0.56)		<.001		0.50 (0.45-0.57)		<.001			0.71 (0.57-0.89)		.003			0.79 (0.67-0.93)		.005			0.36 (0.21-0.62)^c^		<.001
	≥65		0.28 (0.23-0.31)		<.001		0.40 (0.35-0.45)		<.001			0.22 (0.16-0.31)		<.001			0.49 (0.41-0.59)		<.001			N/A		
**In a couple**																								
	Yes		Reference		N/A		Reference		N/A			Reference		N/A			Reference		N/A			Reference		N/A
	No		1.00 (0.92-1.09)		.98		1.44 (1.32-1.56)		<.001			1.27 (1.09-1.49)		.003			0.87 (0.77-0.97)		.01			1.33 (0.96-1.85)		.09
**Professional situation**																								
	Worker		Reference		N/A		Reference		N/A			Reference		N/A			Reference		N/A			Reference		N/A
	Student		1.25 (1.09-1.44)		.002		1.74 (1.49-2.04)		<.001			0.83 (0.64-1.09)		.174			0.73 (0.59-0.89)		<.001			0.80 (0.51-1.26)		<.001
	Job seeker		0.94 (0.76-1.15)		.53		1.14 (0.93-1.39)		.21			0.81 (0.60-1.11)		.193			1.06 (0.82-1.36)		.69			1.02 (0.57-1.81)		.95
	Not employed or retired		0.41 (0.37-0.45)		<.001		0.65 (0.60-0.71)		<.001			0.38 (0.30-0.48)		<.001			0.47 (0.41-0.54)		<.001			0.25 (0.12-0.52)		<.001
**Educational level (ISCED^d^ 2011)**																								
	3		Reference		N/A		Reference		N/A			Reference		N/A			Reference		N/A			Reference		N/A
	≥6		0.96 (0.84-1.09)		.55		0.91 (0.80-1.02)		.11			0.70 (0.55-0.89)		.004			1.58 (1.30-1.91)		<.001			0.46 (0.29-0.73)		<.001
	4-5		1.05 (0.92-1.20)		.45		0.96 (0.84-1.09)		.51			0.68 (0.54-0.87)		.002			1.34 (1.10-1.64)		.004			0.47 (0.29-0.74)		<.001
	1-2		0.64 (0.53-0.78)		<.001		0.55 (0.47-0.65)		<.001			0.67 (0.48-0.70)		.01			1.64 (0.80-1.42)		.69			0.48 (0.25-0.92)		.03
**Access to outdoor space**																								
	Yes		Reference		N/A		Reference		N/A			Reference		N/A			Reference		N/A			Reference		N/A
	No		1.17 (1.07-1.27)		<.001		1.50 (1.38-1.63)		<.001			1.17 (1.01-1.37)		.043			1.04 (0.93-1.16)		.56			1.64 (1.17-2.29)		.004
**Well-being (WEMWBS^e^ score)**																								
	Per 1-point increase		0.96 (0.95-0.96)		<.001		0.96 (0.96-0.96)		<.001			0.96 (0.95-0.97)		<.001			0.96 (0.96-0.97)		<.001			0.96 (0.94-0.98)		<.001
**General stress (0-10 VAS^f^)**																								
	Per 1-point increase		1.15 (1.13-1.17)		<.001		1.12 (1.10-1.14)		<.001			1.14 (1.11-1.18)		<.001			1.12 (1.10-1.15)		<.001			1.09 (1.02-1.16)		.01
**Living space**																								
	Per 5 square meters/person increase		0.95 (0.94-0.96)		<.001		0.98 (0.97-0.99)		<.001			0.97 (0.96-0.99)		<.001			0.96 (0.95-0.97)		<.001			0.96 (0.93-1.00)		.64
**Confined with other people**																								
	Yes		Reference		N/A		Reference		N/A			Reference		N/A			Reference		N/A			Reference		N/A
	No (alone)		0.92 (0.84-1.01)		.09		0.78 (0.72-0.86)		<.001			1.04 (0.88-1.23)		.69			0.77 (0.68-0.88)		<.001			0.95 (0.66-1.37)		.78
**Housing location**																								
	Urban		Reference		N/A		Reference		N/A			Reference		N/A			Reference		N/A			Reference		N/A
	Periurban		1.00 (0.90-1.11)		.95		0.75 (0.68-0.83)		<.001			0.85 (0.69-1.05)		.14			1.01 (0.88-1.16)		.86			0.81 (0.50-1.32)		.41
	Rural		0.91 (0.82-1.01)		.07		0.71 (0.64-0.78)		<.001			0.88 (0.72-1.06)		.18			0.96 (0.85-1.15)		.57			0.81 (0.51-1.28)		.37
**Working conditions**																								
	Telecommuting		Reference		N/A		Reference		N/A			Reference		N/A			Reference		N/A			Reference		N/A
	Not working		0.58 (0.51-0.65)		<.001		1.11 (0.99-1.24)					0.48 (0.39-0.59)		<.001			0.68 (0.58-0.79)		<.001			0.69 (0.43-1.11)		.13
	Working in the workplace		0.90 (0.80-1.02)		.10		1.23 (1.09-1.38)		<.001			0.66 (0.54-0.83)		<.001			0.99 (0.85-1.15)		.86			0.89 (0.56-1.44)		.66
**Psychiatric treatment**																								
	Never		Reference		N/A		Reference		N/A			Reference		N/A			Reference		N/A			Reference		N/A
	Past		1.27 (1.09-1.47)		<.001		1.28 (1.11-1.49)		<.001			1.04 (0.79-1.35)		.81			1.10 (0.90-1.34)		.35			1.13 (0.67-1.90)		.66
	Current		1.94 (1.62-2.31)		.002		1.32 (1.10-1.59)		.003			1.64 (1.22-2.22)		.001			1.77 (1.39-2.26)		<.001			0.99 (0.52-1.85)		.97
**Addiction treatment**																								
	Never		Reference		N/A		Reference		N/A			Reference		N/A			Reference		N/A			Reference		N/A
	Past		1.01 (0.77-1.33)		.95		1.00 (0.78-1.28)		.98			1.48 (1.01-2.15)		.04			1.06 (0.73-1.54)		.77			0.82 (0.38-1.80)		.64
	Current		1.11 (0.68-1.80)		.69		1.04 (0.66-1.66)		.87			1.15 (0.65-2.03)		.65			1.20 (0.63-2.29)		.58			0.62 (0.17-2.19)		.46

^a^OR: odds ratio.

^b^N/A: not applicable.

^c^Due to power requirements, the age categories of 50-64 years and ≥65 years were pooled in the model exploring cannabis use increase.

^d^ISCED: International Standard Classification of Education.

^e^WEMWBS: Warwick-Edinburgh Mental Well-Being Scale.

^f^VAS: visual analog scale.

**Table 3:** Results of the adjusted analyses exploring the increase in caloric/salty food, screen use, and substance use in the early phase of COVID-19 containment in France among the general population.

Characteristic		Caloric/salty food intake (n=10,771; 3233 increase vs 7538 no increase)		Screen use (n=11,267; 7274 increase vs 3993 no increase)			Tobacco use (n=2787; 996 increase vs 1791 no increase)			Alcohol use (n=7108; 1761 increase vs 5347 no increase)			Cannabis use (n=666; 208 increase vs 458 no increase)		
		aOR^a^ (95% CI)	*P* value		aOR (95% CI)	*P* value		aOR (95% CI)	*P* value		aOR (95% CI)	*P* value		aOR (95% CI)	*P* value
**Gender**															
	Male	Reference	N/A^b^		Reference	N/A		Reference	N/A		Reference	N/A		Reference	N/A
	Female	1.62 (1.48-1.77)	<.001		1.31 (1.21-1.43)	<.001		1.31 (1.11-1.55)	.002		1.02 (0.91-1.14)	.76		0.99 (0.67-1.46)	.95
	Other	1.17 (0.64-2.14)	.63		0.66 (0.35-1.25)			0.95 (0.32-2.78)	.93		0.26 (0.08-0.89)	.03		0.45 (0.07-2.74)	.39
**Age (years)**															
	16-29	Reference	N/A		Reference	N/A		Reference	N/A		Reference	N/A		Reference	N/A
	30-49	0.81 (0.71-0.92)	<.001		0.70 (0.61-0.81)	<.001		0.81 (0.64-1.01)	.07		1.18 (1.01 -1.39)	<.001		0.90 (0.58-1.39)	.65
	50-64	0.54 (0.47-0.63)	<.001		0.68 (0.58-0.79)	<.001		0.71 (0.55-0.93)	.01		0.84 (0.69-1.01)	.07		0.48 (0.25-0.89)^c^	.02
	≥65	0.42 (0.34-0.53)	<.001		0.65 (0.53-0.80)	<.001		0.32 (0.20 -0.50)	<.001		0.76 (0.56-1.07)	.10			
**In a couple**															
	Yes	Reference	N/A		Reference	N/A		Reference	N/A		Reference	N/A		Reference	N/A
	No	0.84 (0.74-0.94)	.003		1.18 (1.06-1.32)	<.001		1.30 (1.06-1.59)	.01		0.92 (0.79-1.07)	.27		1.18 (0.77-1.79)	.46
**Professional situation**															
	Worker	Reference	N/A		Reference	N/A		Reference	N/A		Reference	N/A		Reference	N/A
	Student	0.89 (0.75-1.05)	.17		1.17 (0.97-1.42)	.10		0.61 (0.44-0.84)	.003		0.71 (0.56-0.90)	.004		0.55 (0.31-0.95)	.03
	Job seeker	0.86 (0.69-1.08)	.20		0.69 (0.55-0.86)	.001		0.84 (0.59-1.20)	.35		1.02 (0.77-1.36)	.87		0.94 (0.48-1.82)	.86
	No employment/retired	0.72 (0.59-0.87)	<.001		0.51 (0.43-0.61)	<.001		0.68 (0.48-0.97)	.03		0.72 (0.55-0.94)	.02		0.22 (0.09-0.52)	<.001
**Educational** **level (ISCED^d^ 2011)**															
	3	Reference	N/A		Reference	N/A		Reference	N/A		Reference	N/A		Reference	N/A
	≥6	0.87 (0.75-1.00)	.06		0.95 (0.83-1.09)	.46		0.69 (0.53-0.90)	.006		1.52 (1.24-1.86)	<.001		0.38 (0.22-0.65)	<.001
	I4-5	0.94 (0.82-1.09)	.44		0.97 (0.85-1.11)	.65		0.68 (0.53-0.88)	.003		1.25 (1.02-1.54)	.03		0.41 (0.24-0.69)	<.001
	1-2	0.74 (0.60-0.90)	.003		0.60 (0.50-0.71)	<.001		0.72 (0.51-1.02)	.06		1.14 (0.85-1.54)	.39		0.48 (0.23-1.01)	.05
**Access to outdoor space**															
	Yes	Reference	N/A		Reference	N/A		Reference	N/A		Reference	N/A		Reference	N/A
	No	0.95 (0.85-1.05)	.32		1.16 (1.05-1.29)	0.005		1.00 (0.82-1.23)	.98		0.92 (0.80-1.06)	.27		1.54 (1.01-2.38)	.048
**Well-being (WEMWBS^e^ score)**															
	Per 1-point increase	0.98 (0.97-0.98)	<.001		0.98 (0.97-0.98)	<.001		0.97 (0.96-0.98)^c^	<.001		0.97 (0.96-0.98)	<.001		0.96 (0.93-0.98)	<.001
**General stress (0-10 VAS^f^)**															
	Per 1-point increase	1.07 (1.05-1.10)	<.001		1.08 (1.05-1.10)	<.001		1.07 (1.03-1.11)	<.001		1.06 (1.03-1.09)	<.001		1.03 (0.95-1.12)	.46
**Living space**															
	Per 5 square meters/person increase	0.98 (0.97-0.99)	.003		0.99 (0.98-1.01)	>0.99		1.01 (0.98-1.03)	.68		0.99 (0.98-1.00)	.19		1.01 (0.96-1.05)	.86
**Confined with other people**															
	Yes	Reference	N/A		Reference	N/A		Reference	N/A		Reference	N/A		Reference	N/A
	No (alone)	1.29 (1.11-1.49)	<.001		1.15 (1.01-1.32)	.049		0.91 (0.70-1.17)	.46		0.87 (0.72-1.05)	.16		0.90 (0.53-1.54)	.72
**Housing location**															
	Urban	Reference	N/A		Reference	N/A		Reference	N/A		Reference	N/A		Reference	N/A
	Periurban	1.08 (0.96-1.22)	.21		0.88 (0.79-0.98)	.03		0.87 (0.68-1.11)	.26		1.04 (0.89-1.22)	.62		1.21 (0.69-2.14)	.50
	Rural	0.98 (0.86-1.11)	.73		0.82 (0.73-0.92)	<.001		0.95 (0.75-1.21)	.71		1.06 (0.90-1.24)	.53		1.12 (0.63-1.98)	.72
**Working conditions**															
	Telecommuting	Reference	N/A		Reference	N/A		Reference	N/A		Reference	N/A		Reference	N/A
	Not working	0.98 (0.87-1.11)	.72		1.69 (1.48-1.93)	<.001		0.87 (0.70-1.09)	.24		1.00 (0.85-1.18)	.98		0.87 (0.56-1.36)	.56
	Working in the workplace	1.08 (0.95-1.23)	.21		0.83 (0.73-0.94)	.003		1.47 (1.17-1.86)	.001		1.06 (0.90-1.24)	.49		0.98 (0.58-1.65)	.93
**Psychiatric treatment**															
	Never	Reference	N/A		Reference	N/A		Reference	N/A		Reference	N/A		Reference	N/A
	Past	1.27 (1.09-1.47)	<.001		1.07 (0.91-1.25)	.44		0.80 (0.60-1.08)	.15		1.00 (0.81-1.23)	.99		1.01 (0.55-1.86)	.99
	Current	1.94 (1.62-2.31)	.31		0.92 (0.75-1.13)	.45		1.33 (0.94-1.88)	.10		1.44 (1.10-1.88)^d^	.008		1.12 (0.54-2.35)	.78
**Addiction treatment**															
	Never	Reference	N/A		Reference	N/A		Reference	N/A		Reference	N/A		Reference	N/A
	Past	1.01 (0.77-1.33)	.45		0.95 (0.72-1.24)	.70		1.51 (0.99-2.29)	.06		1.01 (0.68-1.49)	.97		0.70 (0.29-1.73)	.45
	Current	1.11 (0.68-1.80)	.89		0.88 (0.53-1.45)	.63		0.98 (0.53-1.82)	.96		0.78 (0.39-1.58)	.49		0.65 (0.15-2.78)	.57

^a^aOR: adjusted odds ratio.

^b^N/A: not applicable.

^c^Due to power requirements, the age categories of 50-64 years and ≥65 years were pooled in the model exploring cannabis use increase.

^d^ISCED: International Standard Classification of Education.

^e^WEMWBS: Warwick-Edinburgh Mental Well-Being Scale.

^f^VAS: visual analog scale.

After adjustment, the respondents who reported increasing their caloric/salty food intake were more likely to be female, to be aged ≤30 years (see Table 3), to have a partner, to be professionally active, a student, or a job seeker, to report a lower score of well-being and a higher score of general stress, to be locked down alone in a reduced space, and to report current or past treatment for psychiatric disorder (for ORs and 95% CIs, see Table 3).

Based on the results of the multivariable logistic regression models, the respondents who reported increasing their screen use were more likely to be female, to be aged <30 years, to have no partner, to be professionally active, to have a relatively high level of education (ie, ISCED 4 or more), to report a lower score of well-being and a higher score of general stress, to be locked down alone, in a city, and with no access to an outdoor space, and to have stopped their professional activity because of the lockdown (for ORs and 95% CIs, see Table 3).

After adjustment, the interviewees who reported increasing their use of tobacco were more likely to be female, to be aged <50 years, to have no partner, to be professionally active or a job seeker, to have a relatively low level of education (ie, ISCED 3 or less), to report a lower score of well-being and a higher score of general stress, and to continue working in the workplace (for ORs and 95% CIs, see Table 3).

After adjustment, the individuals who reported increasing their alcohol use were more likely to be aged 30 to 49 years, to be professionally active or job seekers, to have a high level of education (ie, ISCED 5 or more), to report a lower score of well-being and a higher score of general stress, and to report current treatment for a psychiatric disorder (for ORs and 95% CIs, see Table 3).

After adjustment, the participants who reported increasing their cannabis use were more likely to be aged <50 years, to have a relatively low level of education (ie, ISCED 4 or less), and to live in a dwelling with no access to an outdoor space (for ORs and 95% CIs, see Table 3).

## Discussion

### Principal Results

Overall, we found that increases were much more frequent than decreases for all the habits explored. Moreover, it appears that the use of screens increased greatly; 4125/11,391 (36.21%) of the survey population reported increased screen use, and 1729 (15.18%) noted difficulties in controlling their screen use.

The early impact of the COVID-19 outbreak and the related lockdown thus appear to be associated with substantial increases in the intake of caloric/salty food as well as in screen and substance use among the French population. In animal models, it is well-demonstrated that reducing social connections enhances stress; as a result, increases are observed in both eating and weight [12] as well as in substance use [13,14]. In humans, epidemiological studies on this topic are more limited, as situations of abrupt reduction in social interaction at the population level are relatively uncommon. However, analogies can be made to studies that investigated individuals enrolled in armed forces during conflicts, which revealed that the social interactions of the individuals were dramatically reduced and that their substance use increased in parallel. For example, studies by Lee Robins [15] among US soldiers who were sent to Vietnam in the 1970s found an important reduction in social interactions accompanied by an important increase in the use of opioids by soldiers during their presence in the field; meanwhile, these patterns of use rapidly and almost completely disappeared after the soldiers returned home. A comparison with our findings should be made with caution, as a situation of war is in no way comparable with that of the COVID-19 lockdown. However, the results of our survey are in line with the fact that a substantial reduction of social habits can be associated with enhanced stress and boredom and thus with increases in addiction-related habits.

Furthermore, our findings enlighten both common aspects and singularities between habits in the profiles of respondents who reported increases. Overall, reduced mental well-being and greater overall stress were shared risk factors of increase for all habits. A current or past history of addiction treatment did not appear to impact the observed changes; this is noteworthy because the respondents were supposed to be more vulnerable to stress and were thus expected to increase or relapse in addictive behaviors [6]. More specifically, the typology of respondents who increased their habits substantially differed depending on the habit. Respondents who increased their intake in caloric/salty foods were primarily young women who were living in smaller dwellings and were locked down alone. Previous studies have demonstrated that emotional eating in stressful environments is more common in women [16,17]. It can thus be hypothesized that women who faced the stress induced by the COVID-19 crisis and the related containment coped more by consuming caloric/salty food relative to men. In this context, living alone in a smaller space or reporting past or current treatment for psychiatric disorder can be seen as additional sources of stress.

Survey respondents who reported increasing their screen use were more likely to be female, less than 30 years of age, single, locked down alone, living in an urban area, without access to an outdoor space, and not working. Stress is also a well-demonstrated risk factor of increasing screen use; it is more expressed in younger people, although usually more commonly in men [18,19]. Living alone is another known risk factor for increased screen use [20,21], and this factor was certainly accentuated during the lockdown. Similarly, not working was previously found to be associated with increased internet use [22]. Interestingly, a previous study found that increased use of the internet in a stressful environment was more frequent in young people living in urban areas [23]. This is in line with our findings, which can be explained by a more confined environment in this case. However, fewer respondents who were currently working in the workplace reported increased screen use compared to telecommuting respondents; this also suggests that enhanced screen use is related to telecommuting in some cases.

Among tobacco smokers, the main risk factors for increased smoking were also being female, age less than 50 years, being single, and low level of education. In line with these findings, female gender, younger age, lower socioeconomic status, and psychological distress are the main factors associated with tobacco use [24]. It thus appears that the same risk factors that are associated with tobacco use in general were associated with increasing tobacco use in the case of the COVID-19 lockdown.

The profile of respondents who increased their alcohol use was different, as this increase preferentially affected people aged 30 to 49 years with high levels of education. A possible explanation is that increased use of alcohol may be less stigmatized than that of tobacco or cannabis, as people who use cannabis and tobacco in France are globally younger, have lower income, and are less educated [25,26]. For this reason, alcohol use may have increased more than tobacco or cannabis use among more educated and middle-aged respondents. In line with this hypothesis, respondents who reported increased cannabis use more specifically consisted of very young workers with low levels of education. This reflects the population of regular users of cannabis in France, which is mainly aged less than 30 years [27]. Moreover, while no study has specifically explored this issue in France, international epidemiological studies have revealed that cannabis use is inversely correlated with level of education [28]. In sum, our findings suggest that increased stress and impaired well-being were common risk factors for increases in all types of addiction-related habits during the early phase of the COVID-19 lockdown in France. However, other sociodemographic characteristics and individual features related to lockdown conditions were associated with increases in more specific habits, thus reflecting a specific vulnerability of some parts of the French population with regard to the different habits explored, namely caloric/salty food intake, screen use, or tobacco, alcohol, or cannabis use.

### Limitations

Our study was a web-based survey performed on a convenience sample with no a priori representativeness of the French population. Although our analyses were weighted based on several basic sociodemographic parameters, we cannot exclude the possibility that important parts of the French population were overrepresented or underrepresented, which may have had an impact on our findings. For example, although illiterate people represent a limited part of the French population, we acknowledge that participating in the survey would be difficult for them without external help. Given the context, however, it would be difficult to rapidly set up a study with more thorough methodological features. Despite this, the rate of tobacco smokers in our study (24.47%) was close to that observed in the French population (25.2% in 2018 [29]). Similarly, 62.40% of our sample declared that they used alcohol; meanwhile, the rate of French adults who used alcohol at least once per year in 2018 was 87%, while the rate of adults who used alcohol at least once a week was 49% [30]. However, a gap was found for cannabis use; 5.4% of our sample reported using this substance, whereas the rate of current users in the adult French population is estimated to reach 11% [27]. This gap may result from social desirability bias, which is the tendency to underreport socially undesirable attitudes and behaviors and to overreport more desirable attributes; this bias is more pronounced with illicit substances [31].

Another limitation pertains to what is conveyed under the notion of “screen use,” which can actually involve many habits, such as video gaming, social networking, or teleworking. The interview may thus have lacked precision on this point, and interpreting the participants’ answers may thus have been more difficult. An additional limitation is that the data we analyzed only pertained to the early phase of the lockdown, and it is perfectly possible that several findings reflected short-term adjustment behaviors that may not be durably sustained over the remaining phase of the lockdown. Another limitation is that we did not explore the interrelations in the changes between habits; thus, we did not explore overlaps in terms of affected populations. Finally, the assessment of how individual habits had changed was entirely subjective with no precise quantification in either terms of amounts or time, which limits the accuracy of the data.

### Comparison With Prior Work

To our knowledge, no previous study has assessed the impact of a national COVID-19 containment measure on eating habits, screen use, or substance use. The increases found in our survey were hypothesized in a recent literature report [6]; however, our contribution provides the first data supporting these assumptions.

### Conclusions

The early phase of COVID-19 containment in France was associated with a substantial proportion of survey respondents reporting increased caloric/salty food intake, screen use, and tobacco, alcohol, and cannabis use. The increase was particularly large for screen use, which affected two-thirds of the sample. Furthermore, the profiles of individuals who increased their habits displayed shared features, particularly poorer well-being and increased stress; however, specificities between each type of increase also revealed some populational singularities, particularly related to gender, age category, and level of education. Thus, targeted prevention messages should be developed to address the types of habits to which subcategories of the population are more vulnerable during and after the containment period. 

## Appendix

Multimedia Appendix 1 CHERRIES checklist.

Multimedia Appendix 2 The questionnaire used for the survey (in English).

Multimedia Appendix 3 Flowchart demonstrating how questionnaires were selected for the analyses.

## Abbreviations

CHERRIES: Checklist for Reporting Results of Internet E-Surveys
COVID-19: coronavirus disease
ISCED: International Standard Classification of Education
VAS: visual analog scale
WEMWBS: Warwick–Edinburgh Mental Well-Being Scale
