# Correction: Global Changes and Factors of Increase in Caloric/Salty Food Intake, Screen Use, and Substance Use During the Early COVID-19 Containment Phase in the General Population in France: Survey Study

**DOI:** 10.2196/31906

**Published:** 2021-07-20

**Authors:** Benjamin Rolland, Frédéric Haesebaert, Elodie Zante, Amine Benyamina, Julie Haesebaert, Nicolas Franck

**Affiliations:** 1 Service Universitaire d'Addictologie de Lyon CH Le Vinatier Hospices Civils de Lyon Bron France; 2 Inserm U1028, CNRS UMR5292 Université Claude Bernard Lyon 1 Bron France; 3 Centre Ressource de Réhabilitation Psychosociale CH Le Vinatier Lyon France; 4 PSYCOMADD 4872 Université Paris-Saclay Paris France; 5 CH Le Vinatier Pôle Centre Rive Gauche Bron France; 6 Service de recherche et d’épidémiologie Pôle de santé publique Hospices Civils de Lyon Lyon France

In “Global Changes and Factors of Increase in Caloric/Salty Food Intake, Screen Use, and Substance Use During the Early COVID-19 Containment Phase in the General Population in France: Survey Study” (JMIR Public Health Surveill 2020;6(3):e19630), one error was noted.

In the originally published article, one paragraph in the *Results* section incorrectly referred to “alcohol use” instead of “cannabis use.” The full paragraph was published as follows:

Finally, regarding cannabis use, 620/11,391 (5.44%) participants reported using cannabis. Among the, 263/620 (39.49%) reported that they had not changed their average daily use of alcohol, whereas 162 (24.32%) declared having moderately increased their alcohol use, 46 (6.91%) increased their alcohol use in a difficult-to-control manner, 150 (22.52%) reduced or stopped their alcohol use without craving/withdrawal, and 45 (6.76%) reduced their alcohol use with craving/withdrawal.

This paragraph has been corrected as follows:

Finally, regarding cannabis use, 620/11,391 (5.44%) participants reported using cannabis. Among the, 263/620 (39.49%) reported that they had not changed their average daily use of cannabis, whereas 162 (24.32%) declared having moderately increased their cannabis use, 46 (6.91%) increased their cannabis use in a difficult-to-control manner, 150 (22.52%) reduced or stopped their cannabis use without craving/withdrawal, and 45 (6.76%) reduced their cannabis use with craving/withdrawal.

The correction will appear in the online version of the paper on the JMIR Publications website on July 20, 2021, together with the publication of this correction notice. Because this was made after submission to PubMed, PubMed Central, and other full-text repositories, the corrected article has also been resubmitted to those repositories.

