# Supplementary material for: Global Changes and Factors of Increase in Caloric/Salty Food Intake, Screen Use, and Substance Use During the Early COVID-19 Containment Phase in the General Population in France: Survey Study
Source: JMIR Public Health Surveill. 2020 Sep 18;6(3):e19630. doi: 10.2196/19630 (PMC7505683; doi:10.2196/19630)
Supplement: Multimedia Appendix 1 [file publichealth_v6i3e19630_app1.docx]

**Supplemental Document 1: Checklist for Reporting Results of Internet E-Surveys (CHERRIES)**

| **Item Category** | **Checklist**  **Item** | **Explanation** |
| --- | --- | --- |
| **Design** | Describe survey design | Target population: French General population |
| **IRB approval and informed consent**  **process** | IRB approval | In accordance with the French law, the survey protocol was submitted to, and approved by, the *Commission Nationale Informatique et Libertés* (CNIL): certificate # 2035126 v 0 |
|  | Informed consent | The welcome page of the survey (<https://sondage.inserm.fr/index.php/461237/lang-fr> ) describes the study, including its length, purpose, and how data would be collected and stored. Clicking to participate was an equivalent of a consent. |
|  | Data protection | No personal identifying information was collected. The survey was hosted and all data stored on a secure server. |
| **Development and**  **pre-testing** | Development and  testing | The survey was not pre-tested. However, several parts of it include validated questionnaire, such as the Warwick–Edinburgh Mental Well-Being Scale (WEMWBS) or the visual analog scale for stress. |

| **Recruitment process and description of the sample having access to the questionnaire** | Open survey versus  closed survey | The survey was open to any participant able to follow the instructions. However, in the present study, data of participants aged less than 18 years were not analyzed |
| --- | --- | --- |
|  | Contact mode | The address of the survey portal was diffused on the Web, using social media and press coverage. |
|  | Advertising the survey | The address of the survey portal was diffused on the Web, using social media and press coverage. |
| **Survey**  **administration** | Web/E-mail | The *Centre Ressource Réhabilitation* (CRR) is the survey manager.  Website : <https://centre-ressource-rehabilitation.org/>  email: [_centre_ressource@ch-le-vinatier.fr](mailto:_centre_ressource@ch-le-vinatier.fr) |
|  | Context | The survey platform is hosted by the INSERM, i.e., the National Institute on Health and Medical Research.  <https://www.inserm.fr/en> |
|  | Mandatory/vol  untary | The survey was completely voluntary. |

|  | Incentives | None |
| --- | --- | --- |
|  | Time/Date | The survey is open every week since the second week of the lockdown in France. However, in the present study, only the data covering the first 5 days of the survey were used |
|  | Randomization of items or  questionnaires | Survey items were not randomized |
|  | Adaptive questioning (use of certain items, or only conditionally displayed based on responses to other items) | No adaptive questioning |
|  | Number of Items | The final survey tool contained a total of 80 questions and took an average of 15-30 minutes to complete. |
|  | Number of screens (pages) | The total number of webpages was 15.  Due to the extensive skip patterns, the total number  of pages a given respondent would see could be less than this. |
|  | Completeness check | Each page of the questionnaire has to be completed before moving to the next page is possible |
|  | Review step | Participants were not required to review their responses at survey completion. A “back” button was provided if participants wished to edit their previous answers. |
| **Response rates** | Unique site visitor |  |
|  | View rate |  |

|  | View rate  (Ratio unique site visitors/unique survey visitors) | Unique site visitors of the welcome page of the survey could not be counted |
| --- | --- | --- |
|  | Participation  rate (Ratio unique survey page visitors/agreed  to participate) | Cf. Flow-chart provided in Supplementary Document |
|  | Completion rate (Ratio agreed to participate/finished  survey) | Cf. Flow-chart provided in Supplementary Document |
| **Preventing**  **multiple**  **entries from**  **the same**  **individual** | Cookies used | No cookies were used |
|  | IP check | The same IP address could fill in the questionnaire only once during a same week of survey |
|  | Log file analysis | No Log file analysis |

| **Analysis** | Handling of incomplete questionnaires | Only questionnaires with complete answers were used in the analyses (cf. flow chart) |
| --- | --- | --- |
|  | Questionnaire s  submitted with an atypical timestamp | Time stamps were not assessed, but participants who wished to leave the survey were proposed to receive an email indicating a link to resume the fill-in procedure |
|  | Statistical  correction | Data were weighted based on national sociodemographic data (please see details in the manuscript) |

Eysenbach, G. (2004). Improving the quality of web surveys: the checklist for reporting results of internet e-­‐surveys (cherries). *Journal of medical Internet research*, 6(3)e34 doi:10.2196/jmir.6.3.e34. <http://www.jmir.org/2004/3/e34/>
