# Supplementary material for: Global Changes and Factors of Increase in Caloric/Salty Food Intake, Screen Use, and Substance Use During the Early COVID-19 Containment Phase in the General Population in France: Survey Study
Source: JMIR Public Health Surveill. 2020 Sep 18;6(3):e19630. doi: 10.2196/19630 (PMC7505683; doi:10.2196/19630)
Supplement: Multimedia Appendix 2 [file publichealth_v6i3e19630_app2.pdf]

It will be sent several times at different times during thelockdown period.

Thank you for the time spent to complete this questionnaire, which will be invaluable in better understanding the psychological effects of thecontainment period.

Notes: This questionnaire can be filled into 15-30 minutes and can be completed in several stages. For any difficulty(s) encountered, please contact us via the following address: Elodie.ZANTE@ch-le-vinatier.fr

Section A: socio-demographic factors

The following questions are designed to get to know your situation (outside the lockdwown)

A1. What is your gender?

- male ☐
- female ☐
- other ☐

A2. Are you pregnant?

- yes ☐
- no ☐
- maybe ☐

A3. Are you in the last trimester of your pregnancy?

- Yes ☐
- No ☐

A4. How old are you?

Note an integer number

☐☐☐

A5. What is your marital status?

single ☐
married or as a couple ☐
divorced or separated ☐
widowed ☐

A6. Before the lockdwown, did you live in France (including overseas territories)?

Yes ☐
No ☐

A7. What is your country of residence?

☐☐☐☐☐☐☐☐☐☐☐☐☐☐☐☐☐☐☐☐☐☐☐☐

A8. What is the postal code of your residence (outside this period of lockdown)?

☐☐☐☐☐

A9. Prior the lockdwown, how many people were sharing your residence (including you)?

☐☐

A10. How many children do you have? (whether they live with you or not)

If you have one or more dependent children who are not yours (stepfamily, full-time childcare, etc.), please count them.

Also include your adult child(ren) who has/have left the family home.

☐☐

A11. How old is/are your child(ren)?

if you have only one child, write his or her age in both boxes.

For any child under 1 year old, write 0

youngest ☐☐
eldest ☐☐

A12. Do you have any pets or farm animals (outside the lockdwown period)?

Yes ☐
No ☐

A13. Before the lockdown, what was your work situation?

Employed worker ☐
Self-employed worker / freelancer ☐
In search of a job ☐
Student ☐

No professional activity (e.g. stay-at-home parent,...) ☐

On disability ☐

Retired ☐

A14. What is your socio-professional category?

- farmer or holder ☐
- craftsman or merchant ☐
- executive or senior intellectual profession (excluding health professionals) ☐
- intermediary profession (excluding health professionals) ☐
- medical or paramedical profession ☐
- social worker ☐
- employee (excluding healthcare professionals) ☐
- worker ☐
- Student ☐
- other ☐

A15. What is your profession?

- hospital nursing assistant ☐
- self-employed caregiver ☐
- hospital nurse ☐
- self-employed nurse ☐
- health executive ☐
- medical intern ☐
- hospital doctor ☐
- self-employed doctor ☐
- pharmacist ☐
- kinesiotherapist ☐
- midwife ☐
- dentist ☐
- other health professional ☐

A16. What is your education level?

- No degree ☐
- Middle school ☐
- Other Intermediate Degree ☐
- High School Diploma / A-levels ☐
- Other Higher Education Intermediate Degree ☐
- Bachelor's level ☐
- Master level ☐
- Ph.D. level and above ☐

Section B: Warwick-Edinburgh Mental Well-being Scale (WEMWBS)

The statements below are about your feelings and thoughts. For each statement, tick the box that best corresponds to your experience this past week.

B1.

	None of the time	Rarely	Some of the time	Often	All of the time
I've been feeling optimistic about the future	☐	☐	☐	☐	☐
I've been feeling useful	☐	☐	☐	☐	☐
I've been feeling relaxed	☐	☐	☐	☐	☐
I've been feeling interested in other people	☐	☐	☐	☐	☐
I've had energy to spare	☐	☐	☐	☐	☐
I've been dealing with problems well	☐	☐	☐	☐	☐
I've been thinking clearly	☐	☐	☐	☐	☐
I've been feeling good about myself	☐	☐	☐	☐	☐
I've been feeling close to other people	☐	☐	☐	☐	☐
I've been feeling confident	☐	☐	☐	☐	☐
I've been able to make up my own mind about things	☐	☐	☐	☐	☐
I've been feeling loved	☐	☐	☐	☐	☐
I've been interested in new things	☐	☐	☐	☐	☐
I've been feeling cheerful	☐	☐	☐	☐	☐

Section C: Stress visual numeric scale

C1. On a scale ranging from 0 (total absence of stress) to 10 (maximum imaginable stress), what is your level of stress in these 3 different areas since the beginning of the lockdwown ?

	0	1	2	3	4	5	6	7	8	9	10
stress level at work	☐	☐	☐	☐	☐	☐	☐	☐	☐	☐	☐
personal stress level	☐	☐	☐	☐	☐	☐	☐	☐	☐	☐	☐
overall stress level	☐	☐	☐	☐	☐	☐	☐	☐	☐	☐	☐

C2. On a scale from 0 (total absence of stress) to 10 (maximum imaginable stress), what is your stress level since the beginning of the lockdwown concerning your delivery?

	0	1	2	3	4	5	6	7	8	9	10
level of stress regarding personal and family organization	☐	☐	☐	☐	☐	☐	☐	☐	☐	☐	☐
stress level in relation to the medical follow-up and the delivery process	☐	☐	☐	☐	☐	☐	☐	☐	☐	☐	☐
overall stress level	☐	☐	☐	☐	☐	☐	☐	☐	☐	☐	☐

Section D: history

D1. Before the lockdwown, were you hospitalized?

- Yes ☐
- No ☐

D2. Do you have a chronic illness and/or disability?

- Yes ☐
- No ☐

D3. Have you ever been treated for a psychiatric problem and/or an addiction problem? (by a psychologist/psychiatrist/addiction specialist)

- Yes , currently monitored ☐
- Yes, previously monitored ☐
- No, never ☐

D4. **What type(s) of follow-up(s) do you currently have? What have you had in the past?**

	currently	stopped	never
psychological monitoring	☐	☐	☐
psychiatric care	☐	☐	☐
addictological monitoring	☐	☐	☐

D5. **For which symptom(s) did you consult a psychiatrist and/or psychologist?**

- anxiety / anxiety disorder (generalized anxiety, phobia, OCD...) ☐
- depression ☐
- sleep disorders ☐
- post-traumatic stress disorder ☐
- addictions ☐
- hallucinations, delusions, or psychotic disorder ☐
- eating disorders ☐
- other behavioural disorders ☐
- neurodevelopmental disorders (Autism Spectrum Disorders, Attention Deficit Disorder with or without Hyperactivity...) ☐
- other ☐

D6. **Have you ever been in voluntary or forced lockdwown or isolation before?**

some examples: imprisonment, spiritual retreat, solitary hiking, quarantine, locking oneself up...

- Yes ☐
- No ☐

D7. **What previous lockdwown situation(s) have you experienced and how long did they last?**

	Never	Less than 1 month	From 1 to 6 months	From 6 months to 1 year	More than 1 year
Disease and/or epidemic context	☐	☐	☐	☐	☐
Work/study setting (voluntary)	☐	☐	☐	☐	☐
Work or study context (forced)	☐	☐	☐	☐	☐
Context of imprisonment and/or police custody	☐	☐	☐	☐	☐

	Never	Less than 1 month	From 1 to 6 months	From 6 months to 1 year	More than 1 year
Context of aggression and/or forcible confinement	☐	☐	☐	☐	☐
Natural disaster and/or accident context	☐	☐	☐	☐	☐
Spiritual retreat	☐	☐	☐	☐	☐
A hike or other single-handed sporting activity (sailing...)	☐	☐	☐	☐	☐
Other situation(s)	☐	☐	☐	☐	☐

D8. Outside of this lockdwown period, how often do you have social interactions with family or friends?

	Less than once a week	Once a week	Several times a week	Every day
Directly face to face	☐	☐	☐	☐
On the phone	☐	☐	☐	☐
By SMS	☐	☐	☐	☐
On social networks (Facebook, Instagram...)	☐	☐	☐	☐

Section E: coronavirus

E1. Do you agree with lockdwown measure applied?

- Full agreement ☐
- Fairly agree ☐
- Neither agree nor disagree ☐
- Rather disagree ☐
- Strongly disagree ☐

E2. Are you satisfied with your level of information on the lockdwown?

- Very satisfied ☐
- Pretty satisfied ☐
- Neither satisfied nor dissatisfied ☐
- Rather not satisfied ☐
- Not satisfied at all ☐

E3. **Did you find official informations about the coronavirus sufficiently clear (mode of transmission, prevention...)?**

- Very clear ☐
- Fairly clear ☐
- Neither clear nor fuzzy ☐
- Rather blurry ☐
- Very fuzzy ☐

E4. **Are you in contact with any person(s) likely to be contaminated by the coronavirus?**

Choose the answer that best suits your current situation

- I am lockdwown in my home and I am contaminated ☐
- I am lockdwown in my home without a contaminated person ☐
- I am confined in my home with one or more contaminated person(s) ☐
- I am in direct contact with people or equipment suspected of being contaminated outside the facility ☐
- I am in direct contact with people outside my home (e.g. at work). ☐
- I leave my home but I am not in direct contact with outsiders (on a farm, for example). ☐

E5. **Are you concerned about the lack of access to protective materials (masks, hydroalcoholic gels)?**

- No ☐
- Pretty much no ☐
- Pretty much ☐
- Yes ☐

E6. **You would like to have more access to it for**

- protect you from others outside the home ☐
- protect your loved ones in case you become infected ☐
- be able to help your loved ones protect themselves ☐
- protect yourself and others in the workplace ☐
- have something to protect you later on if someone around you becomes infected ☐

E7. **Are you currently concerned about access to basic necessities?**

- No, I'm not worried about that ☐
- Yes, I'm concerned, but I currently have access to everything I need ☐
- Yes, I'm worried and I currently don't have access to everything I need ☐

Section F: the lockdwown

F1. **During the lockdwown period, do you live in France (including overseas territories)?**

- Yes ☐
- No ☐

F2. **Where are you lockdwown ? (country)**

F3. **Are you lockdwown in your usual accommodation (the one you occupy outside the confinement period)?**

- yes ☐
- No, I'm confined to another dwelling ☐
- No, I'm in the hospital ☐
- No, I don't have a fixed address (homeless) ☐

F4. **What is the postal code of your dwelling during this lockdwown period ? (or your city if you are currently homeless or hospitalized)**

F5. **Where are you hospitalized during this lockdwown period ?**

in a public care service (hospital, CHU...)

- in a public care service (hospital, CHU...) ☐
- in a private care unit ☐
- in a public psychiatric ward ☐
- in a private psychiatric ward (clinic) ☐
- other ☐

F6. **What is the surface area of your dwelling during this lockdwown period (in m2)?**

Divide an area in feet by 10 to convert it to a square (m2) (approximation)

Please note a number of m2 of living space (excluding exteriors)

☐☐☐

F7. **During the lockdwown, do you have access to:**

- a balcony ☐
- a terrace ☐
- a privative garden ☐
- a private swimming pool ☐
- sports equipment (e.g. treadmill, exercise bike, etc...) ☐
- a private sports field ☐
- none of this ☐

F8. **During the lockdwown, do you you live:**

- in an urban area ☐
- in a suburban area ☐
- in a rural area ☐

F9. **How many people share your accommodation during the lockdwown (including you)?**

☐☐

F10. **Are your children lockdwown with you in the same accommodation?**

- Yes ☐
- No ☐

F11. **How many of your children are lockdwown with you in the same accommodation?**

☐☐

F12. **If you have minor children, does the lockdwown affect custody of your children ?**

- Yes ☐
- No ☐

F13. **Are there any members of your family lockdwown with you, excluding your children?**

- Yes ☐
- No ☐

F14. How many people live with you, excluding your children?

Count all the people who share your roof (including your possible spouse, your possible roommate(s), etc.) excluding your possible child(ren).

☐☐

F15. Did you change your delivery schedule during the lockdwown?

- yes ☐
- no ☐

F16. What change(s) has/have been decided since the start of the lockdwown?

- Place of delivery ☐
- Arrangements for transport to the place of delivery ☐
- Follow-up dates of your pregnancy ☐
- How to monitor your pregnancy ☐
- The organization of the family around your delivery ☐
- Other change(s) ☐

F17. Are you working during the lockdwown?

- Yes, I'm teleworking ☐
- yes, I'm on my work site exclusively ☐
- yes, I'm on my work site and teleworking. ☐
- No, I'm on short-time work ☐
- No, I'm on medical leave ☐
- no, I'm on leave or on childcare leave ☐
- No, I'm looking for a job ☐

F18. What is your workload during the lockdwown period?

- less than before containment ☐
- same as before containment ☐
- higher than before containment ☐
- highly variable and unpredictable ☐

F19. Are you afraid to go outside to work during the lockdwown?

- No ☐
- Pretty much no ☐
- Pretty much yes ☐
- Yes ☐

F20. Is the lockdwown going to have a financial impact on your budget?

- Very likely ☐
- Probably ☐
- Probably not ☐
- Certainly not ☐

F21. Will the lockdwown put you in a precarious situation?

- Very likely ☐
- Probably ☐
- Probably not ☐
- Certainly not ☐

F22. During the lockdwown, on average over the week, how much time per day do you spend on each of these activities?

	0	less than 30 minutes	from 30 minutes to 1 hour	from 1 to 2 hours	from 2 to 3 hours	more than 3 hours
workand/or study	☐	☐	☐	☐	☐	☐
take care of yourself (wash, dress, do your hair and make-up...)	☐	☐	☐	☐	☐	☐
talk to your family and friends	☐	☐	☐	☐	☐	☐
care for children	☐	☐	☐	☐	☐	☐
clean up / tidy up	☐	☐	☐	☐	☐	☐
do DIY / interior work	☐	☐	☐	☐	☐	☐
cook	☐	☐	☐	☐	☐	☐
getting out of your home (outside of work)	☐	☐	☐	☐	☐	☐
watch the news	☐	☐	☐	☐	☐	☐
read	☐	☐	☐	☐	☐	☐
do creative activities (music, drawing...)	☐	☐	☐	☐	☐	☐
work out	☐	☐	☐	☐	☐	☐
play video games	☐	☐	☐	☐	☐	☐
play with family and friends (online or at home)	☐	☐	☐	☐	☐	☐
social networking	☐	☐	☐	☐	☐	☐

	0	less than 30 minutes	from 30 minutes to 1 hour	from 1 to 2 hours	from 2 to 3 hours	more than 3 hours
phone	☐	☐	☐	☐	☐	☐
text	☐	☐	☐	☐	☐	☐
surf the web	☐	☐	☐	☐	☐	☐
meditate or relax	☐	☐	☐	☐	☐	☐
let the thought wander	☐	☐	☐	☐	☐	☐
ruminating/being the object of anxious fears	☐	☐	☐	☐	☐	☐
take a nap	☐	☐	☐	☐	☐	☐

F23. During the lockdwown, do you have any difficulty with:

- maintain a satisfactory sleep/wake rhythm (sleep at regular hours...) ☐
- fall asleep ☐
- feeling rested ☐
- eat at least 2 meals a day ☐
- don't snack more than you're used to ☐
- establishing new routines ☐
- no particular difficulty ☐

F24. What helps you to cope with the lockdwown ?

- what people around you are saying ☐
- what the media say ☐
- the conviction of a favourable outcome ☐
- the advancement of knowledge and scientific progress ☐
- religious faith ☐
- other non-religious beliefs (magic thoughts, clairvoyance...) ☐
- past experiences and your ability to cope with difficulties ☐
- stories of similar experiences of others who have made it through ☐
- mutual aid and the implementation of collective actions ☐
- the possible beneficial repercussions that containment can have on the planet (on pollution, nature ...) ☐
- possible positive repercussions of individual confinement (lower petrol prices, teleworking, place of living...) ☐
- the use of substances such as alcohol or cannabis ☐

the use of medicines ☐

none of these proposals ☐

Other ☐

Other

F25. During the lockdwown, how did your consumption / use evolve?

Comparez avec la période précédent le confinement

	No use of it	No change	Decrease or cessation without craving/withdrawal	Decrease or cessation with craving/with drawal	Increase (moderate)	Increase (difficult-to-control)
coffee, tea and/or energy drinks	☐	☐	☐	☐	☐	☐
caloric, fatty, sweet and/or salty foods	☐	☐	☐	☐	☐	☐
tobacco	☐	☐	☐	☐	☐	☐
alcohol	☐	☐	☐	☐	☐	☐
cannabis	☐	☐	☐	☐	☐	☐
other drugs (ecstasy, heroin...)	☐	☐	☐	☐	☐	☐
anxiolytics (diazepam, alprazolam...)	☐	☐	☐	☐	☐	☐
painkillers (paracetamol, tramadol...)	☐	☐	☐	☐	☐	☐
sleeping pills	☐	☐	☐	☐	☐	☐
screens (TV, smartphone, tablet, computer ...)	☐	☐	☐	☐	☐	☐

F26. During the lockdwown, how often do you have social interactions with family or friends who are not with you?

	Less than once a week	Once a week	Several times a week	Every day
directly face-to-face	☐	☐	☐	☐
on the phone	☐	☐	☐	☐
via SMS	☐	☐	☐	☐
on social networks (Facebook, Instagram, etc...)	☐	☐	☐	☐

F27. Do you have support during the lockdwown?

Yes ☐

No ☐

F28. Whose support do you have?

- [ ] of person(s) lockdwown with me under the same roof
- [ ] my family (apart from people living under the same roof)
- [ ] my friends (apart from people living under the same roof)
- [ ] my colleagues
- [ ] my neighbours / acquaintances
- [ ] medical or social professionals
- [ ] associations and/or volunteers
- [ ] other source(s) of support

F29. Do you have your pet(s) with you during the lockdwown ?

- [ ] yes
- [ ] no
- [ ] not all

Thank you for taking the time to answer all these questions. We wish you good luck for this period.
