# Supplementary material for: Global Changes and Factors of Increase in Caloric/Salty Food Intake, Screen Use, and Substance Use During the Early COVID-19 Containment Phase in the General Population in France: Survey Study
Source: JMIR Public Health Surveill. 2020 Sep 18;6(3):e19630. doi: 10.2196/19630 (PMC7505683; doi:10.2196/19630)
Supplement: Multimedia Appendix 3 [file publichealth_v6i3e19630_app3.docx]

**Supplemental Document 3**

Flow chart detailing the selection of questionnaires included in the analyses

20,135

Unique ID responses

8,393

Incomplete questionnaires

11,737 complete and operable questionnaires

5 inoperable questionnaires (outliers)

11,742 complete questionnaires

346 questionnaires from others countries

Or age <15 y

11,391 included questionnaires
